# Supplementary material for: Scalable continuous evolution for the generation of diverse enzyme variants encompassing promiscuous activities
Source: Nat Commun. 2020 Nov 6;11:5644. doi: 10.1038/s41467-020-19539-6 (PMC7648111; doi:10.1038/s41467-020-19539-6)
Supplement: Supplementary file 1 — Supplementary Information [file 41467_2020_19539_MOESM1_ESM.pdf]

Supplementary Information for:

Scalable, continuous evolution for the generation of diverse enzyme variants encompassing promiscuous activities

Gordon Rix[1], Ella J. Watkins-Dulaney[2], Patrick J. Almhjell[3], Christina E. Boville[2,#], Frances H. Arnold[2,3], and Chang C. Liu[1,4,5,*]

[1]Department of Molecular Biology and Biochemistry, University of California, Irvine, CA, USA
[2]Division of Biology and Biological Engineering, California Institute of Technology, Pasadena, CA, USA
[3]Division of Chemistry and Chemical Engineering, California Institute of Technology, Pasadena, CA, USA
[4]Department of Biomedical Engineering, University of California, Irvine, CA, USA
[5]Department of Chemistry, University of California, Irvine, CA, USA
[#]Present address: Aralez Bio, Emeryville, CA, USA
[*]ccl@uci.edu

Supplementary Tables

Supplementary Table 1 | Summary of all cultures passaged for evolution of *Tm*TrpB variants.

TrpB variant (strain)	culture volume (mL)	total number passaged	total number successful
**wt *Tm*TrpB (GR-Y053)**	3	4	1
	100	2	2
***Tm*Triple (GR-Y055)**	3	8	2
	100	4	4
***Tm*TripleQ90* (GR-Y057)**	3	8	1
	100	0	0

Supplementary Table 2 | Mutation summary statistics for OrthoRep-evolved TrpB populations.

		non-synonymous mutations			synonymous mutations	
**variant set**	**total number of sequences**	**total unique mutations**	**mean**	**standard deviation**	**mean**	**standard deviation**
consensus	10	43	5.6	2.3	3.0	2.3
1	16	85	8.7	2.1	6.3	4.4
2	60	194	9.3	2.8	6.5	3.0

Supplementary Table 3 | Mutations and identification information for all individual *Tm*TrpB sequences. Note that variant names are abbreviations of the evolution experiment from which the variant was harvested. For example, the variant name WT-100-1-A refers to the arbitrarily designated unique clone A that came from replicate 1 of the 100 mL evolution experiment that started from wt *Tm*TrpB. Likewise, Q90*-003-1-B refers to the arbitrarily designated unique clone B that came from replicate 1 of the 3 mL evolution experiment that started from *Tm*TripleQ90*. Likewise, Tri-003-2-A refers to the arbitrarily designated unique clone A that came from replicate 2 of the 3 mL evolution experiment that started from *Tm*Triple. (See Supplementary Data 4 for mutations and sequences.)

variant set	variant name	number of non-synonymous mutations	number of synonymous mutations	starting sequence
**1**	WT-100-1-A	13	3	TmTrpB
**1**	WT-100-2-A	9	7	TmTrpB
**1**	WT-003-1-A	7	1	TmTrpB
**1**	Q90*-003-1-A	6	11	TmTripleQ90*
**1**	Q90*-003-1-B	11	15	TmTripleQ90*
**1**	Tri-003-1-A	9	3	TmTriple
**1**	Tri-003-1-B	11	4	TmTriple
**1**	Tri-003-1-C	9	3	TmTriple
**1**	Tri-003-2-A	9	6	TmTriple
**1**	Tri-100-1-A	10	5	TmTriple
**1**	Tri-100-2-A	9	7	TmTriple
**1**	Tri-100-2-B	8	6	TmTriple
**1**	Tri-100-3-A	5	3	TmTriple
**1**	Tri-100-4-A	10	1	TmTriple
**1**	Tri-100-4-B	6	14	TmTriple
**1**	Tri-100-4-C	7	11	TmTriple
**2**	WT-100-1-B	14	9	WT TmTrpB
**2**	WT-100-1-C	12	7	WT TmTrpB
**2**	WT-100-1-D	12	9	WT TmTrpB
**2**	WT-100-1-E	14	10	WT TmTrpB
**2**	WT-100-1-F	16	11	WT TmTrpB
**2**	WT-100-2-B	7	9	WT TmTrpB
**2**	WT-100-2-C	7	5	WT TmTrpB
**2**	WT-100-2-D	9	8	WT TmTrpB
**2**	WT-100-2-E	8	9	WT TmTrpB
**2**	WT-100-2-F	9	10	WT TmTrpB
**2**	WT-100-2-G	7	6	WT TmTrpB
**2**	WT-100-2-H	9	9	WT TmTrpB
**2**	WT-100-2-I	10	5	WT TmTrpB
**2**	WT-003-1-B	6	2	WT TmTrpB

**2**	WT-003-1-C	7	5	WT TmTrpB
**2**	WT-003-1-D	7	7	WT TmTrpB
**2**	WT-003-1-E	10	8	WT TmTrpB
**2**	WT-003-1-F	8	4	WT TmTrpB
**2**	WT-003-1-G	9	4	WT TmTrpB
**2**	WT-003-1-H	8	4	WT TmTrpB
**2**	WT-003-1-I	8	6	WT TmTrpB
**2**	Q90*-003-1-C	10	6	TmTripleQ90*
**2**	Q90*-003-1-D	11	9	TmTripleQ90*
**2**	Q90*-003-1-E	14	8	TmTripleQ90*
**2**	Q90*-003-1-F	14	8	TmTripleQ90*
**2**	Q90*-003-1-G	16	6	TmTripleQ90*
**2**	Q90*-003-1-H	12	9	TmTripleQ90*
**2**	Tri-003-1-D	9	4	TmTriple
**2**	Tri-003-1-E	7	5	TmTriple
**2**	Tri-003-1-F	12	3	TmTriple
**2**	Tri-003-1-G	10	5	TmTriple
**2**	Tri-003-1-H	15	6	TmTriple
**2**	Tri-003-1-I	6	4	TmTriple
**2**	Tri-003-2-B	8	11	TmTriple
**2**	Tri-003-2-C	13	4	TmTriple
**2**	Tri-003-2-D	12	8	TmTriple
**2**	Tri-003-2-E	10	3	TmTriple
**2**	Tri-100-1-B	5	5	TmTriple
**2**	Tri-100-1-C	6	5	TmTriple
**2**	Tri-100-1-D	9	6	TmTriple
**2**	Tri-100-1-E	10	8	TmTriple
**2**	Tri-100-1-F	7	2	TmTriple
**2**	Tri-100-1-G	6	5	TmTriple
**2**	Tri-100-1-H	8	4	TmTriple
**2**	Tri-100-2-C	8	5	TmTriple
**2**	Tri-100-2-D	10	15	TmTriple
**2**	Tri-100-2-E	13	10	TmTriple
**2**	Tri-100-2-F	9	10	TmTriple
**2**	Tri-100-2-G	9	15	TmTriple
**2**	Tri-100-2-H	10	10	TmTriple
**2**	Tri-100-2-I	13	4	TmTriple
**2**	Tri-100-3-B	7	3	TmTriple
**2**	Tri-100-3-C	6	3	TmTriple
**2**	Tri-100-3-D	8	4	TmTriple
**2**	Tri-100-3-E	6	2	TmTriple

**2**	Tri-100-3-F	5	3	TmTriple
**2**	Tri-100-4-D	8	8	TmTriple
**2**	Tri-100-4-E	7	7	TmTriple
**2**	Tri-100-4-F	6	6	TmTriple
**2**	Tri-100-4-G	6	5	TmTriple

Supplementary Table 4 | Kinetic parameters of selected *Tm*TrpB variants at 30 °C.

variant	k_{cat} [95% credible region] (s^{-1})	K_M [95% credible region] (μM)	k_{cat}/K_M [95% credible region] (mM^{-1} s^{-1})
***Tm*Triple**	0.2 [0.16, 0.31]	41.23 [14.32, 192.66]	4.89 [1.54, 12.12]
**WT-003-1-A**	0.53 [0.49, 0.58]	3.89 [1.85, 7.99]	137.22 [70.29, 276.04]
**Q90*-003-1-A**	0.77 [0.72, 0.83]	5.79 [3.82, 8.8]	133.38 [91.24, 193.71]
**Tri-100-2-A**	0.62 [0.59, 0.66]	5.58 [3.99, 7.91]	111.89 [81.52, 152.25]

Supplementary Figures

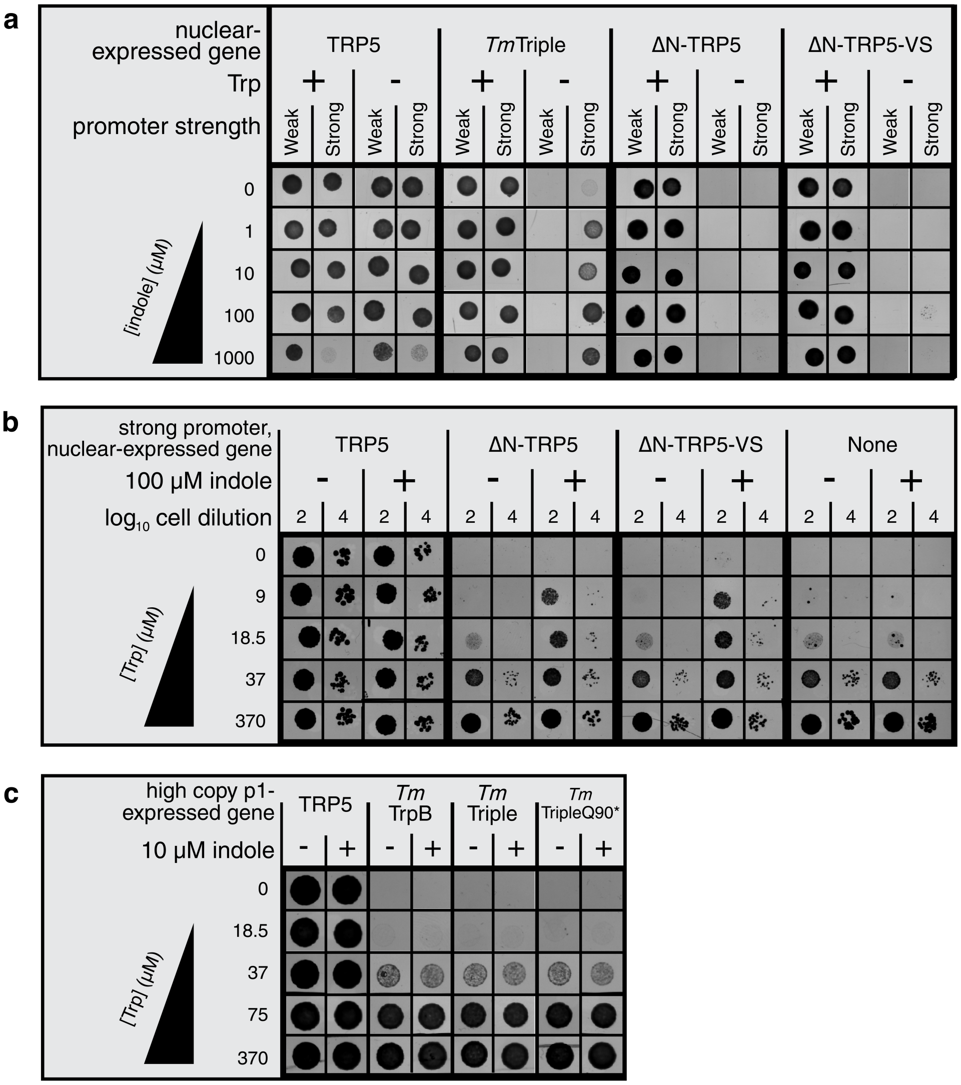

Supplementary Fig. 1 | Evaluation of indole-dependent TRP5 complementation of TrpB variants. a-c, Spot plating assays for Δ*trp5* yeast strains expressing TrpB variants from a nuclear plasmid under two different promoter strengths (weak, pREV1; strong, pTDH3) (**a**), from a nuclear plasmid under a strong promoter (**b**), or from p1 at a high copy number (wt TP-DNAP1 expressed *in trans*) (**c**) grown on indicated growth medium. ΔN-TRP5, N-terminally truncated yeast TRP5 constituting only the region of TRP5 homologous to *Tm*TrpB. ΔN-TRP5-VS, ΔN-TRP5 with two of the three *Tm*Triple mutations relative to wt *Tm*TrpB. The markedly reduced growth at 1000 µM indole only when TRP5 is expressed by a strong promoter may be explained by indole toxicity induced by additional indole production by TRP5.

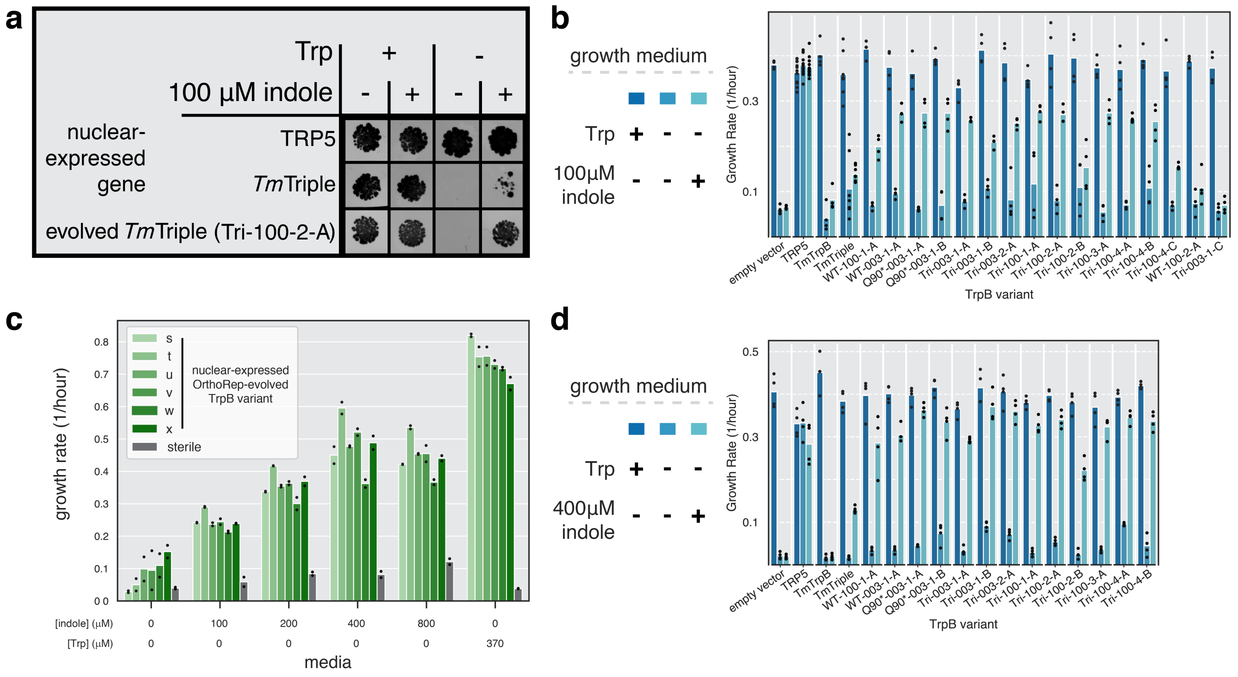

Supplementary Fig. 2 | *In vivo* Trp production by evolved TrpBs. a, Spot plating assay for TRP5-deleted yeast with a nuclear plasmid expressing TRP5, *Tm*Triple, or an individual OrthoRep-evolved *Tm*TrpB variant driven by a promoter (pRNR2) that approximates expression of *Tm*TrpB variants from OrthoRep's p1 plasmid, grown on indicated media. **b-d**, Evaluation of TRP5 complementation by evolved variants through a growth rate assay. Maximum growth rates during exponential growth phase (when rate is above ~0.15 per hour) over a 24-hour period for *Δtrp5* yeast strains transformed with a nuclear plasmid expressing the indicated *Tm*TrpB variant, grown in the indicated growth medium. Points represent growth rate for individual replicates, bars represent the mean growth rate for all replicates**.** Note that growth rates below ~0.15 per hour correspond to cultures that did not enter exponential phase; in these cases, the reported growth rate is not meaningful and instead can be interpreted as no quantifiable growth. **b,** Low indole growth rate test. TRP5 tested in n=13 biologically independent replicates over 5 independent experiments. *Tm*Triple tested in n=8 biologically independent replicates over 4 independent experiments. All other variants tested in n=4 biologically independent replicates in a single experiment. **c**, Optimization of indole concentration. All conditions tested in technical duplicate, although exact sequences of *Tm*TrpB variants were not determined, as the sole purpose of these variants was to evaluate the effect of indole concentration. **d**, High indole growth rate test. A subset of this data is shown in **Fig. 2a**. All variants tested in n=4 biologically independent replicates in a single experiment. Source data are provided as a Source Data file.

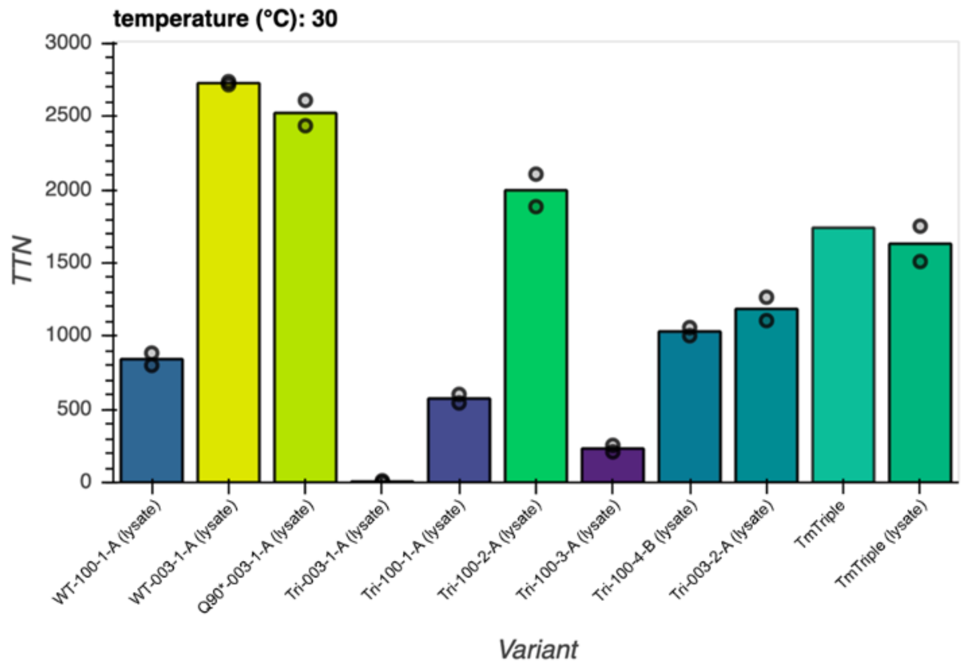

Supplementary Fig. 3 | *In vitro* Trp production by evolved TrpBs with heat treated lysate. Trp production at 30 °C by indicated *Tm*TrpB variants. Reactions with *Tm*Triple were performed with both heat treated lysate (1 hour incubation at 75 °C) and with purified protein, while all other reactions were performed only with heat treated lysate. TTN, total turnover number. Maximum TTN is 5,000. Points represent TTN for individual biological replicates, bars represent mean TTN for reactions with replicates, or TTN for a single replicate otherwise. Reaction with purified *Tm*Triple performed in a single replicate, and all lysate reactions performed in n=2 biologically independent replicates. Source data are provided as a Source Data file.

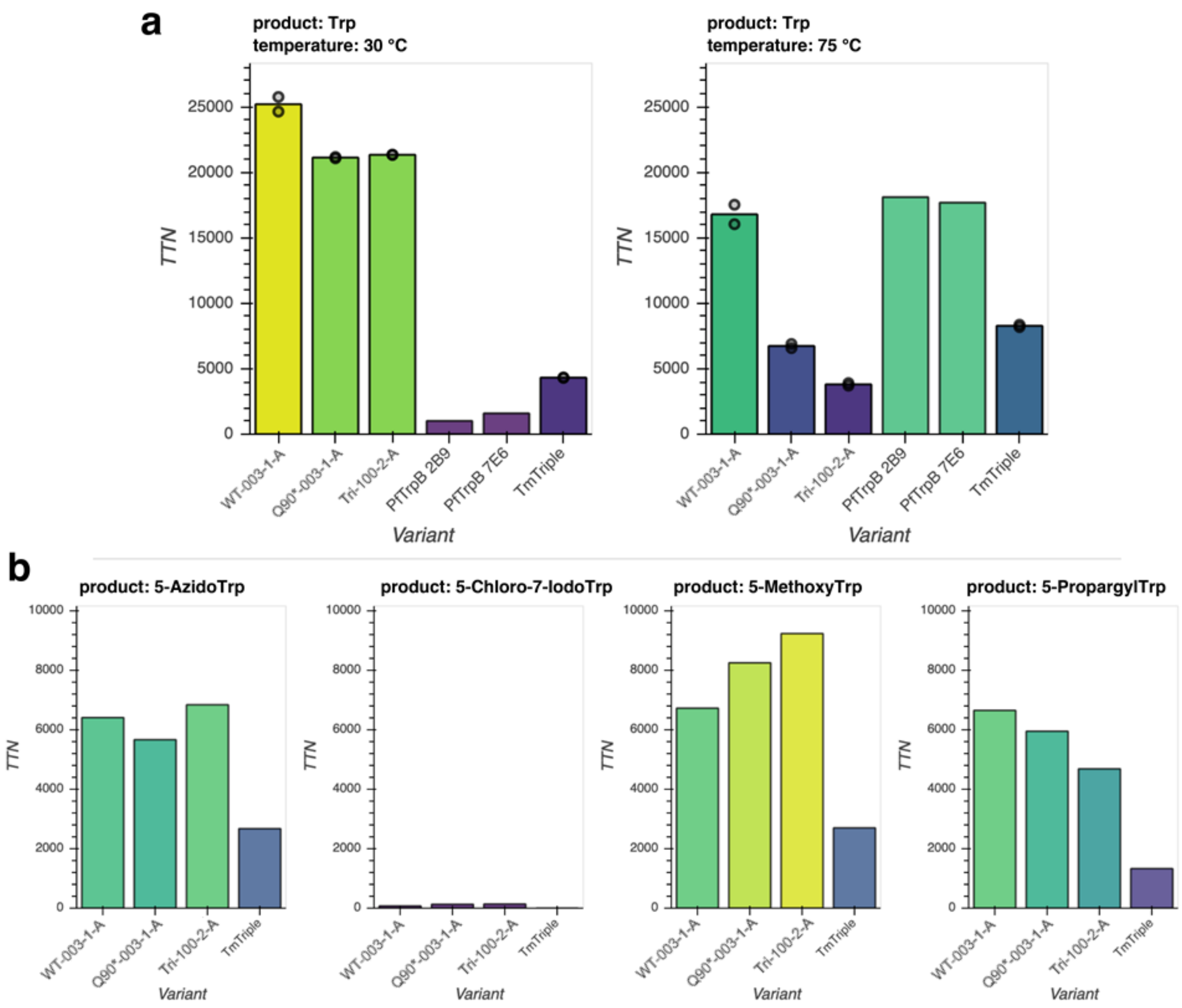

Supplementary Fig. 4 | *In vitro* Trp and Trp analog production with purified enzyme. a,**b**, Production of (**a**) Trp at 30 °C or 75 °C with 40,000 maximum TTN or (**b**) indicated Trp analogs at 30 °C by column purified *Tm*TrpB variants. TTN, total turnover number with 10,000 as maximum TTN. Points represent TTN for independent biological replicates (n=2), bars represent mean TTN for reactions with replicates, or TTN for a single replicate otherwise. Source data are provided as a Source Data file.

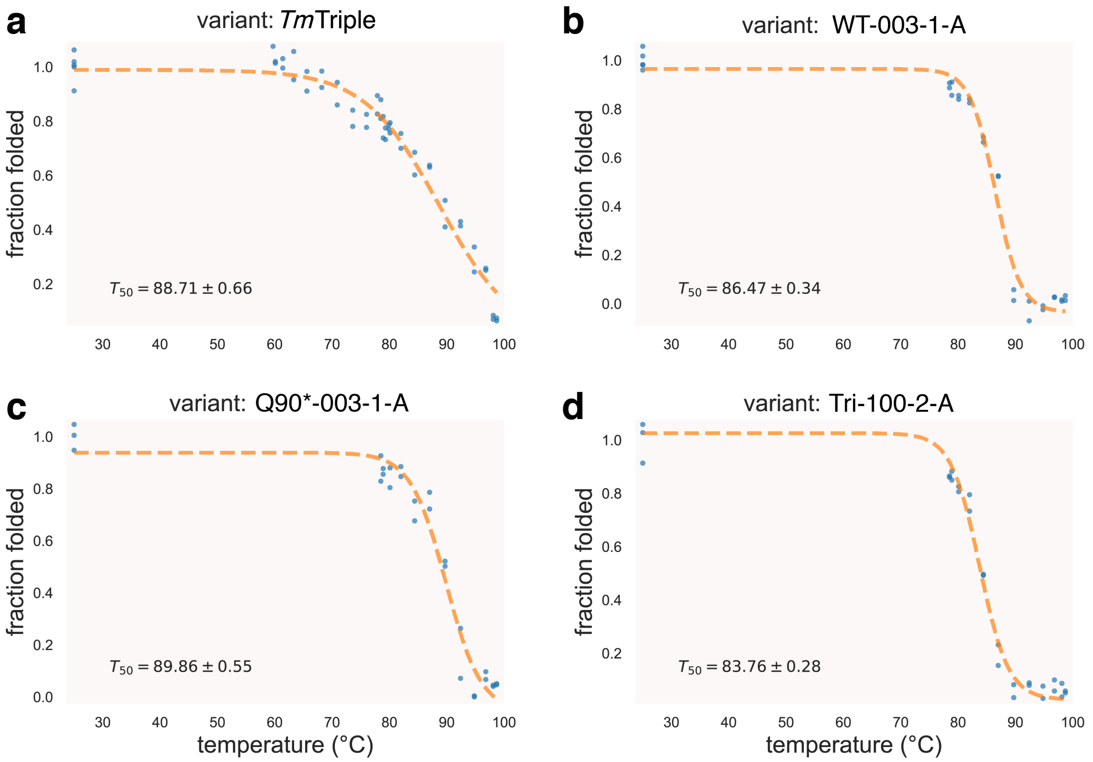

Supplementary Fig. 5 | Thermal shift assay on various *Tm*TrpBs. Proportion of *Tm*TrpB variants TmTriple (**a**), WT-003-1-A (**b**), Q90*-003-1-A (**c**), or Tri-100-2-A (**d**) that remain folded after incubation at indicated temperature for 1 hour, as measured by the fraction of Trp production relative to incubation at 25 °C. T_{50}, temperature at which 50% of enzyme is irreversibly inactivated, as estimated by best fit logistic model (dotted line). Each temperature tested in technical duplicate.

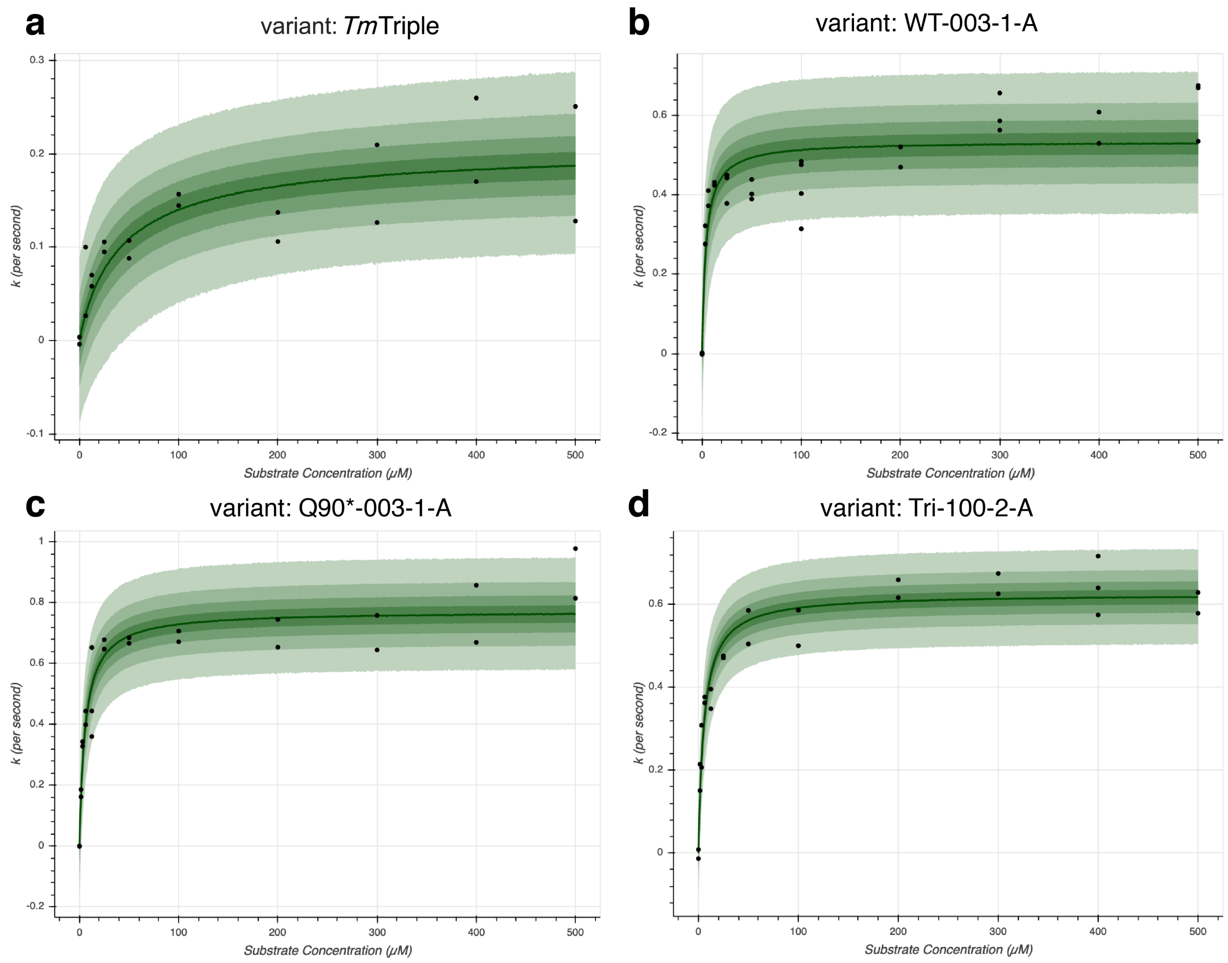

Supplementary Fig. 6 | Michaelis-Menten plots for rate of Trp production at saturating serine for evolved *Tm*TrpB variants. Initial rate of Trp formation (*k*, per second) with TrpB variants *Tm*Triple (**a**), WT-003-1-A (**b**), Q90*-003-1-A (**c**), or Tri-100-2-A (**d**) at saturating serine concentration (40 mM) vs. indole concentration. Points, median estimates for initial rate based on absorbance change over time (see **Methods**). For all four variants, the median estimated Michaelis-Menten curve is shown as a dark green line, with the 25, 50, 75, and 95% credible regions displayed from dark to light green, respectively. Measurements performed in n=3 technical replicates for all substrate concentrations for WT-003-1-A and 400 μM for Tri-100-2-A, and n=2 technical replicates for all other substrate concentrations and samples.

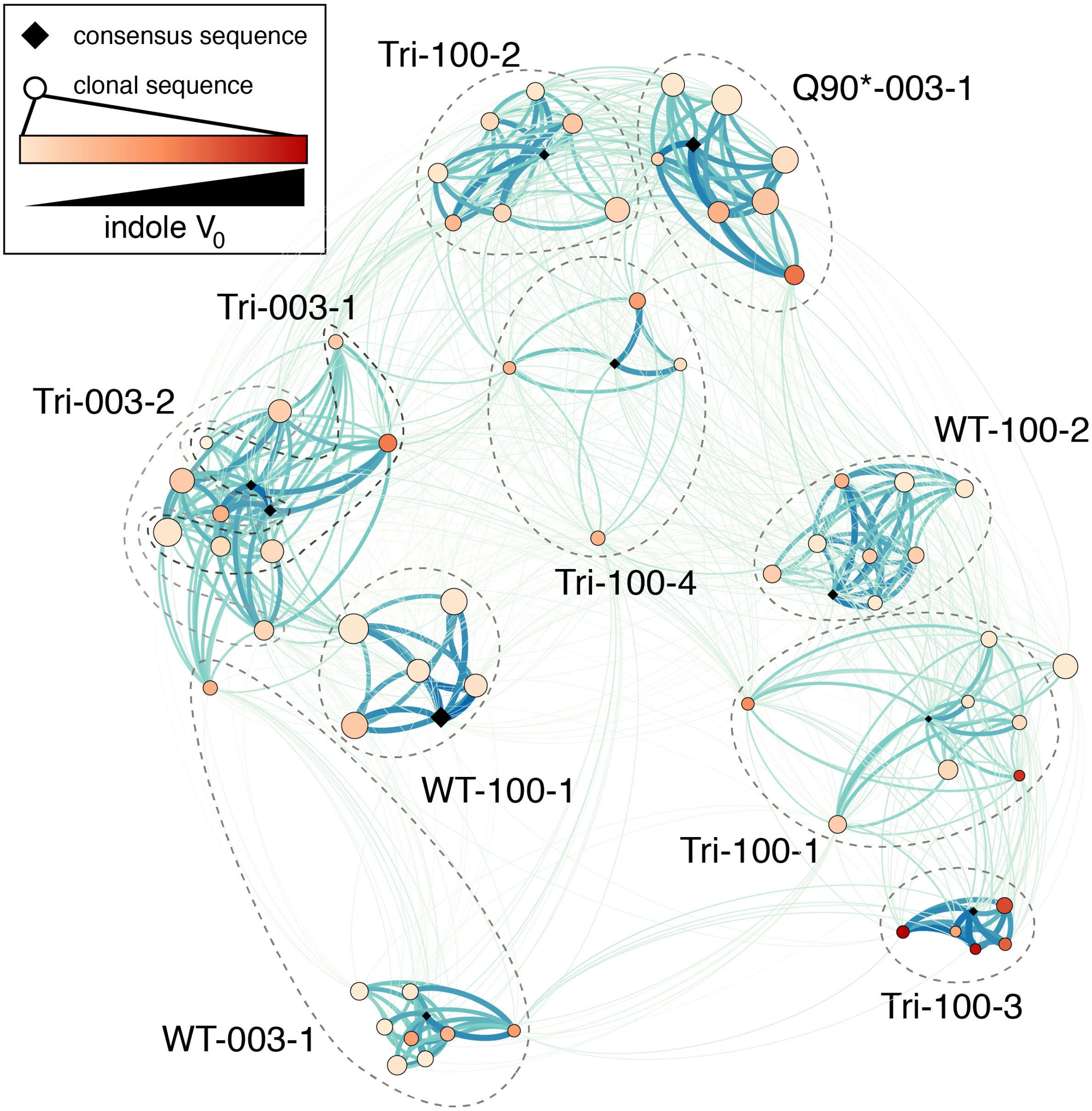

Supplementary Fig. 7 | Relatedness of TrpB panel sequences generated by OrthoRep evolution. Force directed graph where each node represents an individual sequence (all variants from set 2, and variants WT-003-1-A, Q90*-003-1-A, and Tri-100-2-A) or consensus sequence for one of the ten evolved populations. Edge weights are proportional to the number of shared mutations between two nodes. Higher edge weight yields a stronger attractive force between two nodes, and is visualized as a darker color and a thicker line. Nodes for individual sequences are colored according to initial rate of Trp formation, similar to **Fig. 3b**, and are sized according to the number of mutations in the sequence. Dotted lines are drawn around consensus sequences and individual sequences that are derived from the same evolved culture, if nodes are sufficiently clustered to allow it. Graph was visualized using Gephi version 0.9.2.

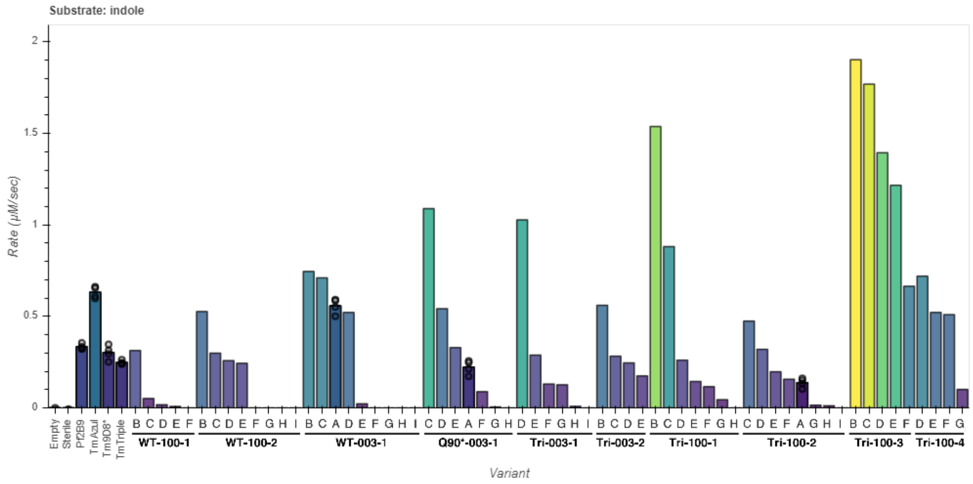

Supplementary Fig. 8 | TrpB panel indole activity by initial rate of Trp formation. Initial rate of Trp formation at saturating L-serine by UV-vis spectrophotometry. Points represent rate for individual replicates, bars represent mean rate for reactions with multiple replicates, or rate for a single replicate otherwise. OrthoRep-evolved variants are ordered first by the population from which they were derived, then by indole activity. Empty, expression vector without TrpB variant. Sterile, reaction master mix without heat treated lysate added. *Pf*2B9, *Tm*Azul, *Tm*9D8*, *Tm*Triple, WT-003-1-A, Q90*-003-1-A, and Tri-100-2-A all performed in n=4 biologically independent replicates; Empty performed in n=3 biological replicates; Sterile performed in n=2 technical replicates; all other reactions performed a single replicate. Source data are provided as a Source Data file (data are identical to those shown in Fig. 3 panel B).

a

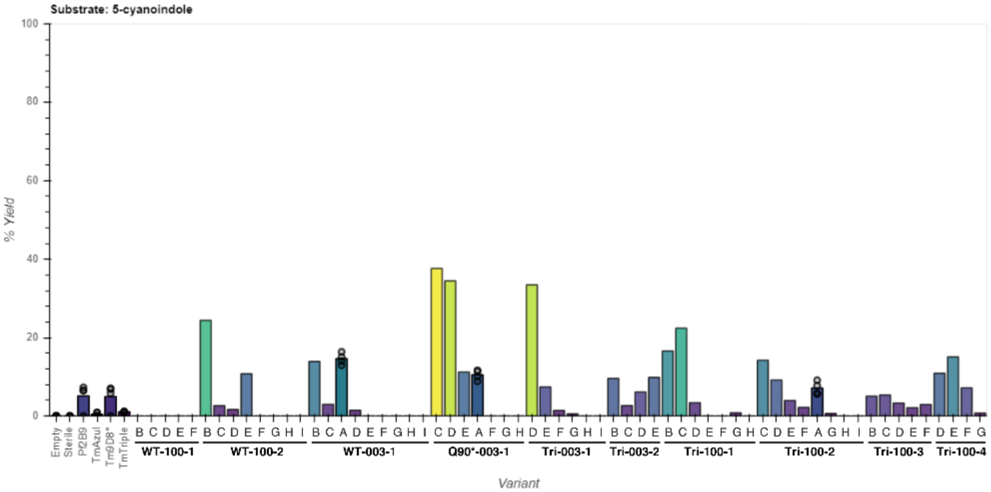
Substrate: 5-cyanoindole
% Yield
100
80
60
40
20
0
Empty
Sterile
Pf2B9
TmAzul
Tm9D8*
TmTriple
WT-100-1
WT-100-2
WT-003-1
Q90*-003-1
Tri-003-1
Tri-003-2
Tri-100-1
Tri-100-2
Tri-100-3
Tri-100-4
Variant

b

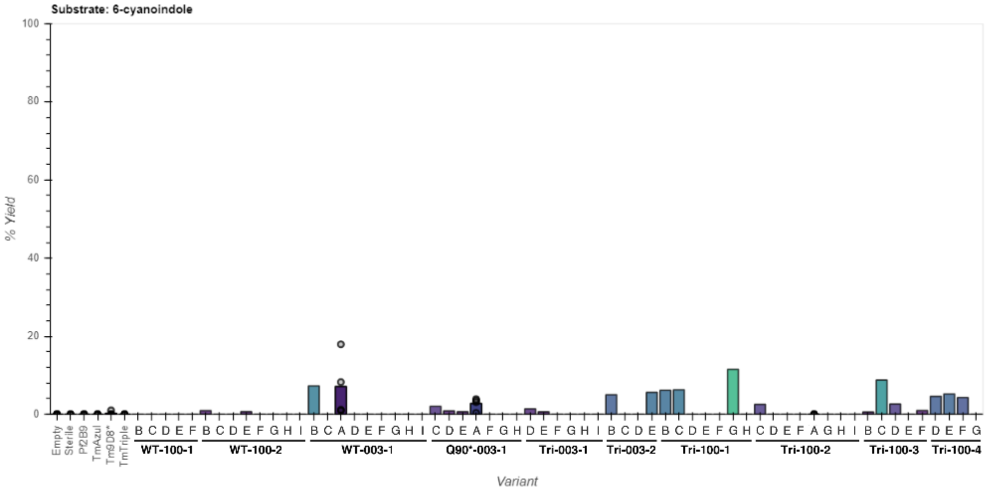
Substrate: 6-cyanoindole
% Yield
100
80
60
40
20
0
Empty
Sterile
Pf2B9
TmAzul
Tm9D8*
TmTriple
WT-100-1
WT-100-2
WT-003-1
Q90*-003-1
Tri-003-1
Tri-003-2
Tri-100-1
Tri-100-2
Tri-100-3
Tri-100-4
Variant

c

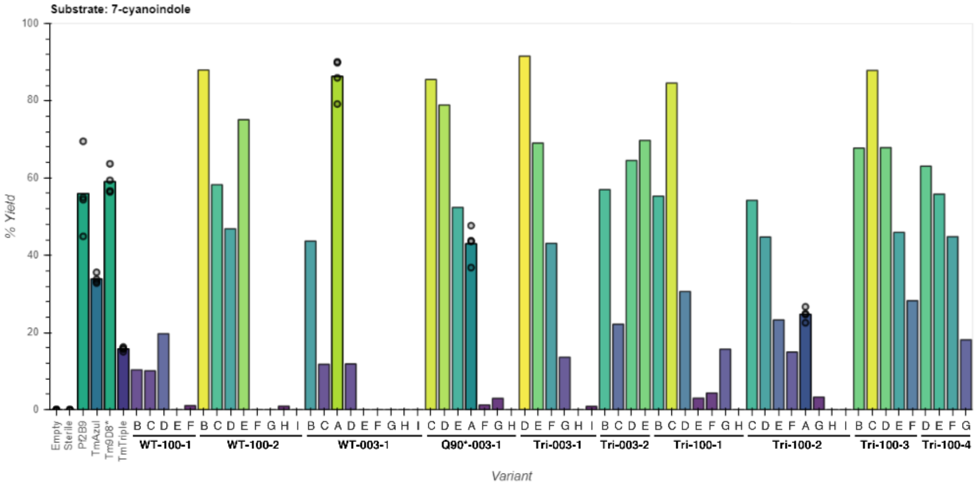
Substrate: 7-cyanoindole
% Yield
100
80
60
40
20
0
Empty
Sterile
Pf2B9
TmAzul
Tm9D8*
TmTriple
WT-100-1
WT-100-2
WT-003-1
Q90*-003-1
Tri-003-1
Tri-003-2
Tri-100-1
Tri-100-2
Tri-100-3
Tri-100-4
Variant

d

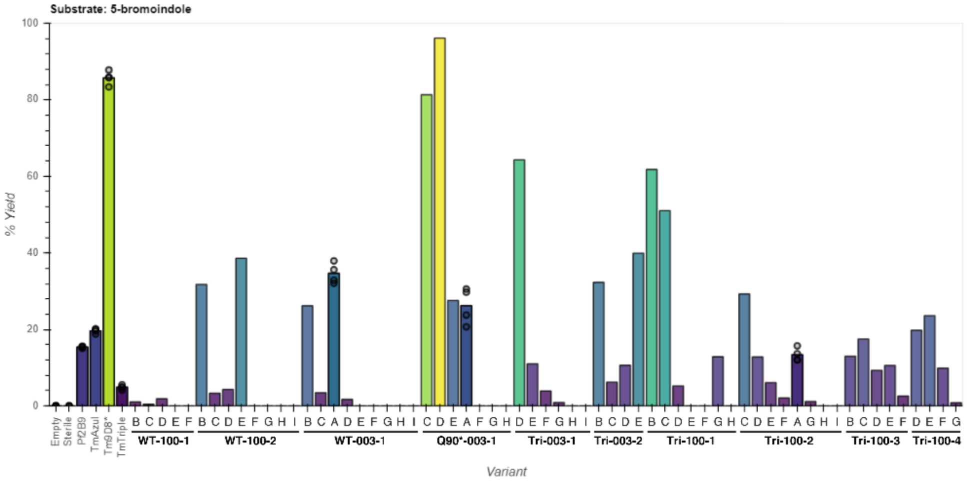
Substrate: 5-bromoindole
% Yield
Variant
Empty
Sterile
Pf2B9
TmAzul
Tm9D8*
TmTriple
WT-100-1
WT-100-2
WT-003-1
Q90*-003-1
Tri-003-1
Tri-003-2
Tri-100-1
Tri-100-2
Tri-100-3
Tri-100-4

e

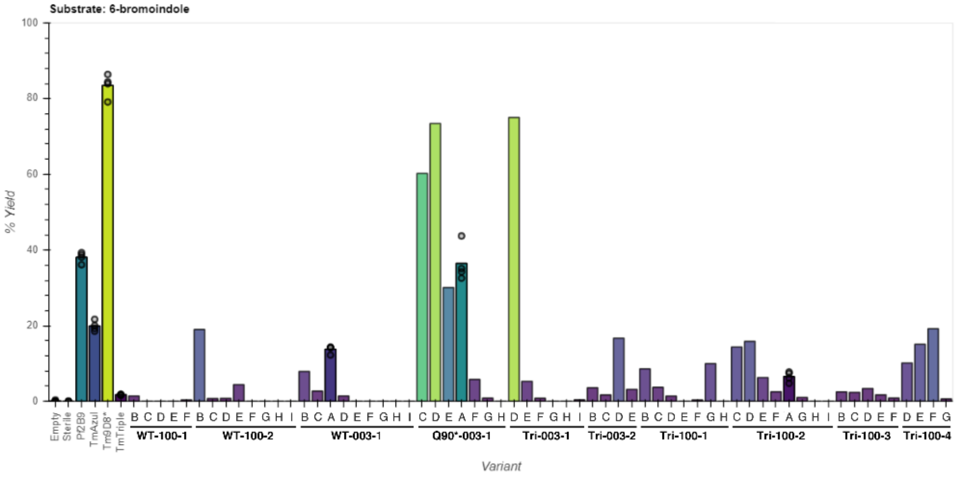
Substrate: 6-bromoindole
% Yield
Variant
Empty
Sterile
Pf2B9
TmAzul
Tm9D8*
TmTriple
WT-100-1
WT-100-2
WT-003-1
Q90*-003-1
Tri-003-1
Tri-003-2
Tri-100-1
Tri-100-2
Tri-100-3
Tri-100-4

f

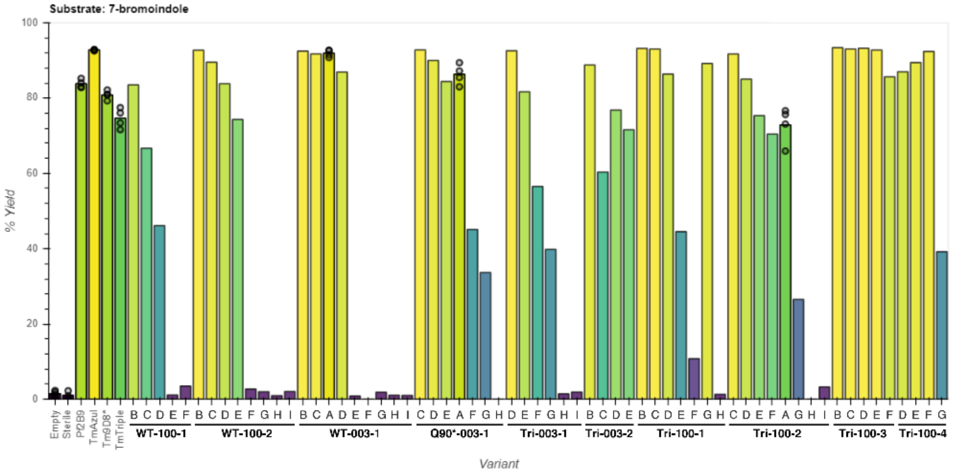
Substrate: 7-bromoindole
% Yield
Variant
Empty
Sterile
Pf2B9
TmAzul
Tm9D8*
TmTriple
WT-100-1
WT-100-2
WT-003-1
Q90*-003-1
Tri-003-1
Tri-003-2
Tri-100-1
Tri-100-2
Tri-100-3
Tri-100-4

g

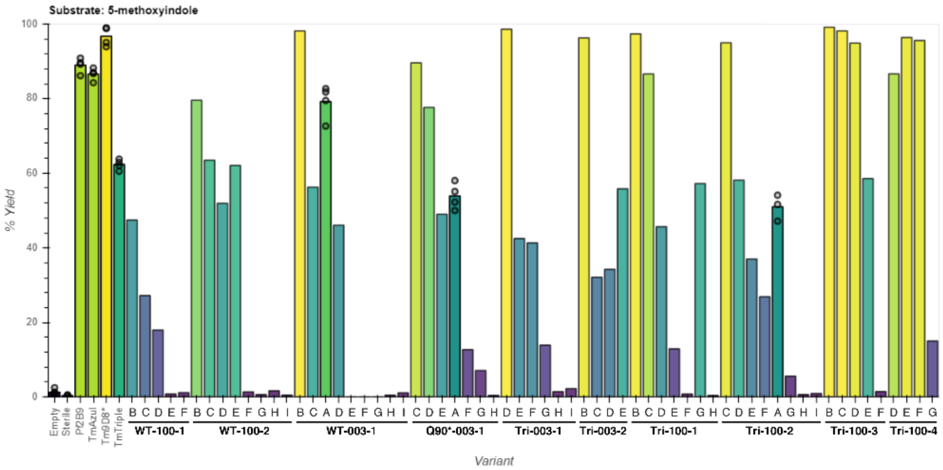
Substrate: 5-methoxyindole
100
80
60
40
20
0
% Yield
Empty
Sterile
Pf2B9
TmAzul
Tm9D8*
TmTriple
WT-100-1
WT-100-2
WT-003-1
Q90*-003-1
Tri-003-1
Tri-003-2
Tri-100-1
Tri-100-2
Tri-100-3
Tri-100-4
Variant

h

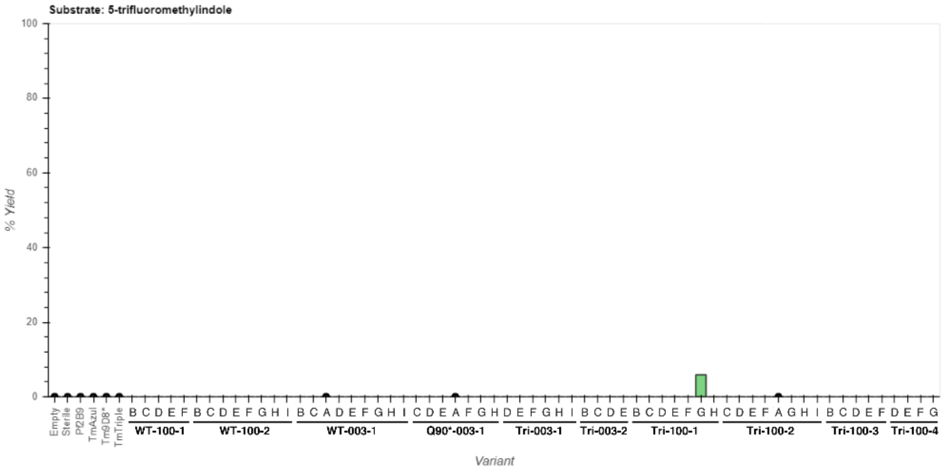
Substrate: 5-trifluoromethylindole
100
80
60
40
20
0
% Yield
Empty
Sterile
Pf2B9
TmAzul
Tm9D8*
TmTriple
WT-100-1
WT-100-2
WT-003-1
Q90*-003-1
Tri-003-1
Tri-003-2
Tri-100-1
Tri-100-2
Tri-100-3
Tri-100-4
Variant

i

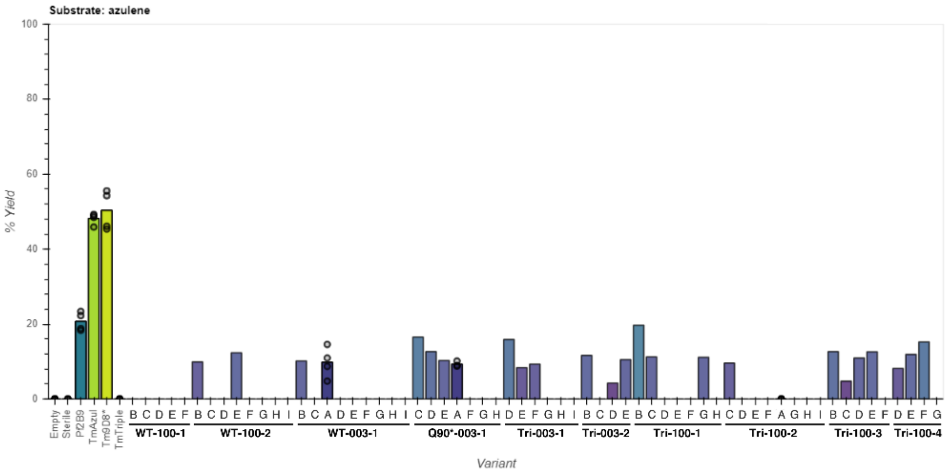

Supplementary Fig. 9 | TrpB panel activity with indole analogs by HPLC yield. a-i, HPLC yield of (**a**) 5-cyanoTrp, (**b**) 6-cyanoTrp, (**c**) 7-cyanoTrp, (**d**) 5-bromoTrp, (**e**) 6-bromoTrp, (**f**) 7-bromoTrp, (**g**) 5-methoxyTrp, (**h**) 5-trifluoromethylTrp, and (**i**) β-(1-azulenyl)-L-alanine (azulene) for indicated variants supplied with L-serine and each indole substrate. Points represent % yield for individual replicates, bars represent mean % yield for reactions with replicates, or % yield for a single replicate otherwise. Replicates and variant order are as in Fig. S8, and populations from which OrthoRep-evolved variants were derived are annotated. Empty, *Pf*2B9, *Tm*Azul, *Tm*9D8*, *Tm*Triple, WT-003-1-A, Q90*-003-1-A, and Tri-100-2-A performed in n=4 biologically independent replicates; sterile performed in n=2 technical replicates; all other reactions performed a single replicate. Source data are provided as a Source Data file (data are identical to those shown in Fig. 3 panel B).

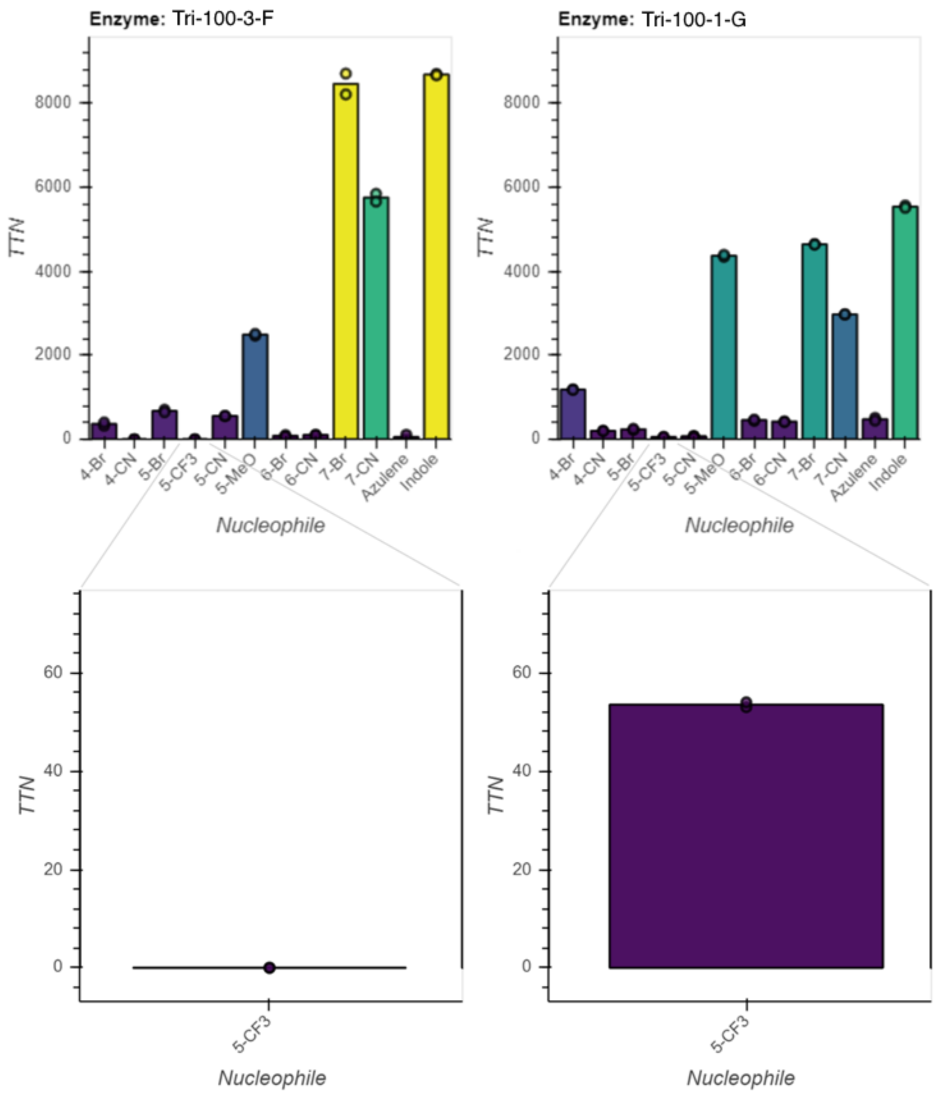

Supplementary Fig. 10 | Substrate activity profiles for large scale purification of variants Tri-100-3-F and Tri-100-1-G. Total turnover number (TTN) for *Tm*TrpB variants Tri-100-3-F and Tri-100-1-G purified at large scale (see **Methods**) and supplied with L-serine and the indicated indole analog, azulene, or indole (nucleophile), with a maximum TTN of 10,000. All reactions were performed in technical duplicate. Points represent TTN for individual replicates, bars represent mean for two replicates. Insets, TTN for 5-trifluoromethylindole with y-axis scale adjusted for clarity. Source data are provided as a Source Data file.

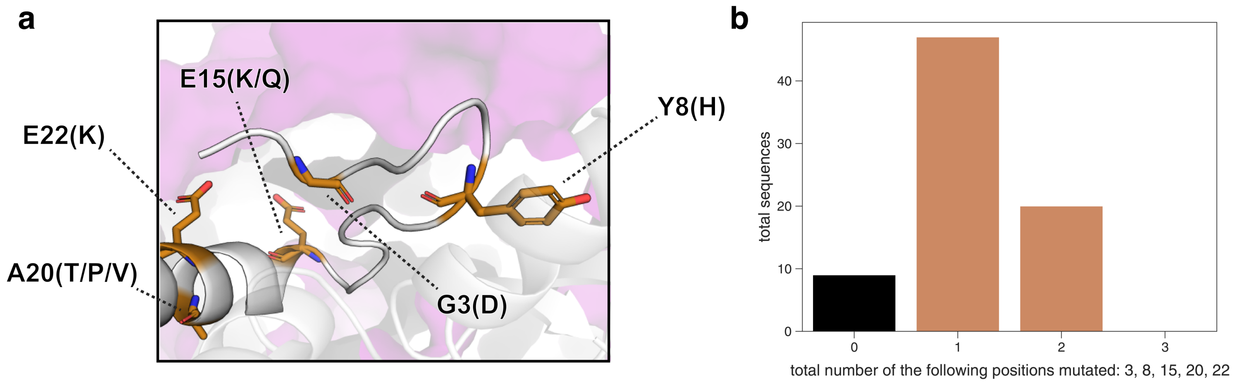

Supplementary Fig. 11 | Commonly observed mutations at the α-subunit interaction interface. a, Homology-predicted *Tm*TrpB structure (based on engineered stand-alone *Pf*TrpB, PDB 6AM8), with commonly mutated *Tm*TrpB residues located near the TrpA interaction interface highlighted. Solvent-exposed regions of TrpA (purple) (PDB 1WDW) are shown as a surface. Mutations are indicated by the wt residue and position, followed by any residues to which this wt residue is mutated in OrthoRep-evolved TrpB sequences. **b**, Total number of sequences in both variant sets 1 and 2 that contain the indicated number of mutations to any of the residues highlighted in panel **a**.

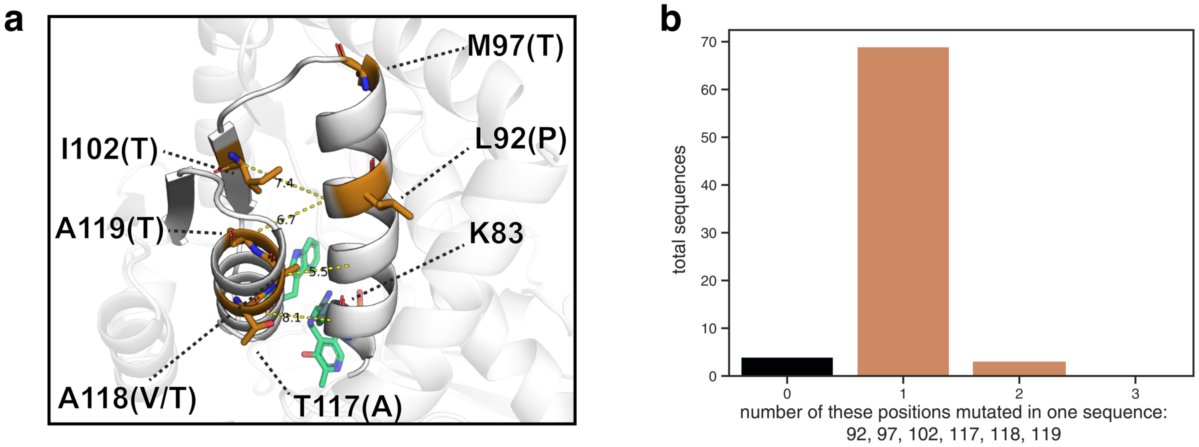

Supplementary Fig. 12 | Commonly observed mutations to residues near a catalytic α-helix. **a**, Homology-predicted *Tm*TrpB structure (aligned to engineered stand-alone *Pf*TrpB, PDB 6AM8) with wt residues on or near the α-helix housing K83, which are commonly mutated in OrthoRep evolved populations (orange). PLP (green) and Trp (green) are shown as sticks, and the catalytic lysine K83 (teal) is shown as spheres. Mutations are indicated by the wt residue and position, followed by any residues to which this wt residue is mutated in OrthoRep-evolved TrpB sequences. Dotted lines connect the α-carbon of residues not located on the K83 α-helix with the α-carbon of the nearest residue on the K83 α-helix, with the distance noted in Ångstroms. **b**, Total number of sequences in both variant sets 1 and 2 that contain the indicated number of mutations to any of the residues highlighted in panel **a**.

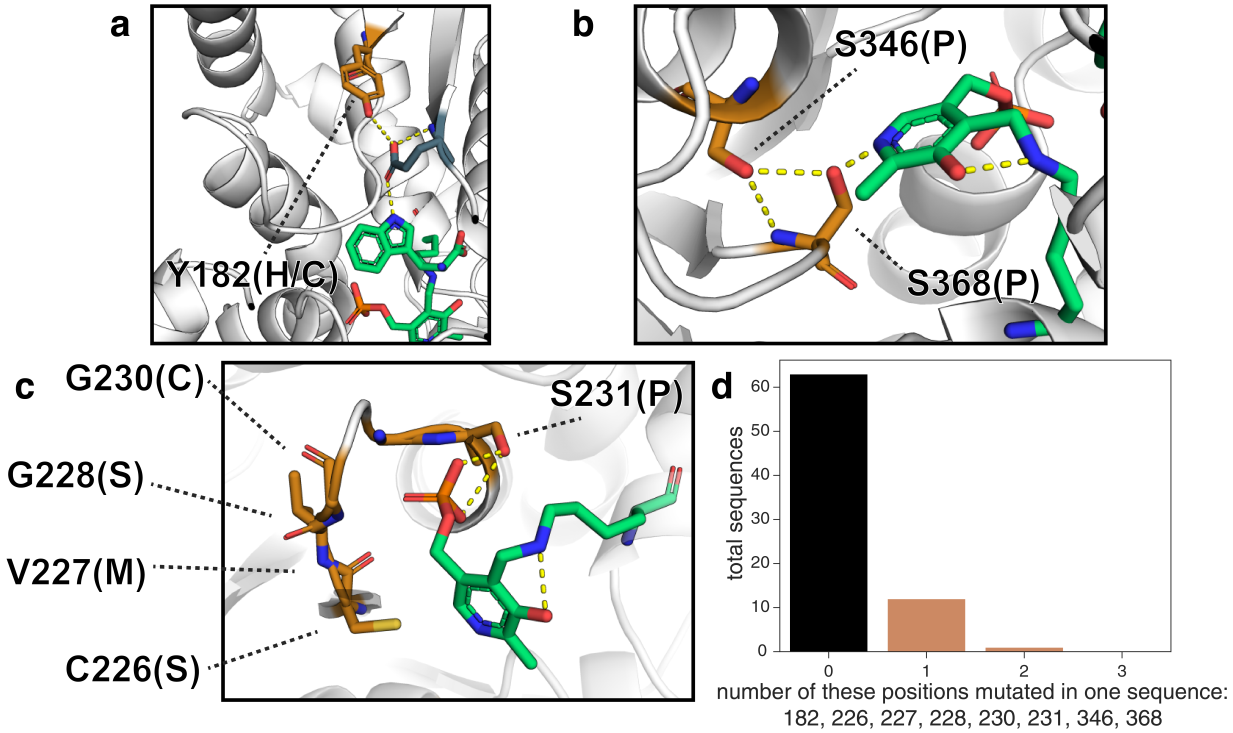

Supplementary Fig. 13 | First- and second-shell active site mutations. a-c, Homology model of *Tm*TrpB (aligned to *Pf*TrpB, PDB: 6AM8) highlighting residues mutated in OrthoRep-evolved variants (orange) that may influence (**a**) indole charge, (**b**) PLP six-member ring binding, and (**c**) PLP-phosphate binding. Mutations are indicated by the wt residue and position, followed by any residues to which this wt residue is mutated in OrthoRep-evolved TrpB sequences. **d**, Total number of sequences in both variant sets 1 and 2 that contain the indicated number of mutations to any of the residues highlighted in panels **a**, **b**, or **c**.

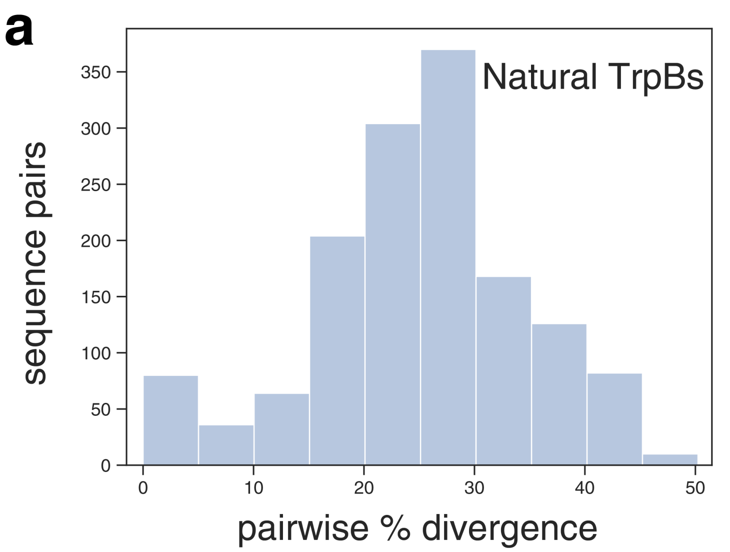

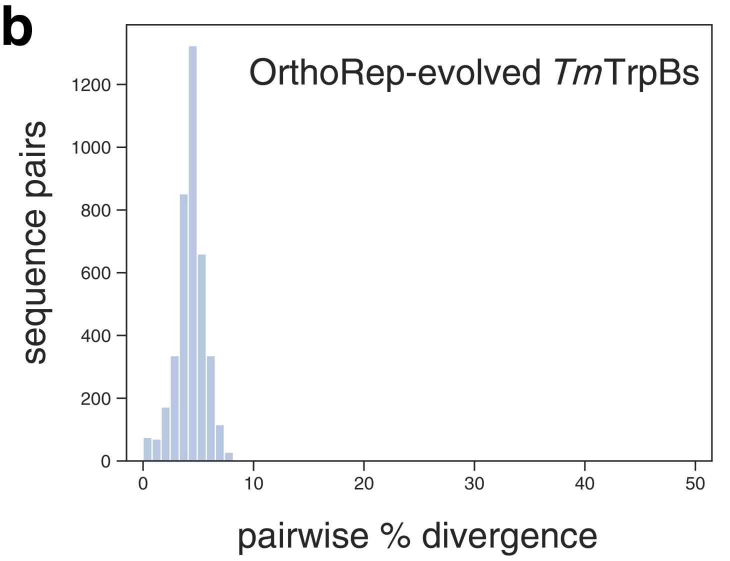

Supplementary Fig. 14 | Sequence divergence for natural and OrthoRep-evolved TrpBs. a-b, Distributions of pairwise % amino acid sequence divergence for a diverse group of 38 naturally occurring mesophilic TrpB variants (**a**) and OrthoRep-evolved variant sets 1 and 2 (**b**). Multiple sequence alignments used to generate these histograms are provided as a Source Data file.

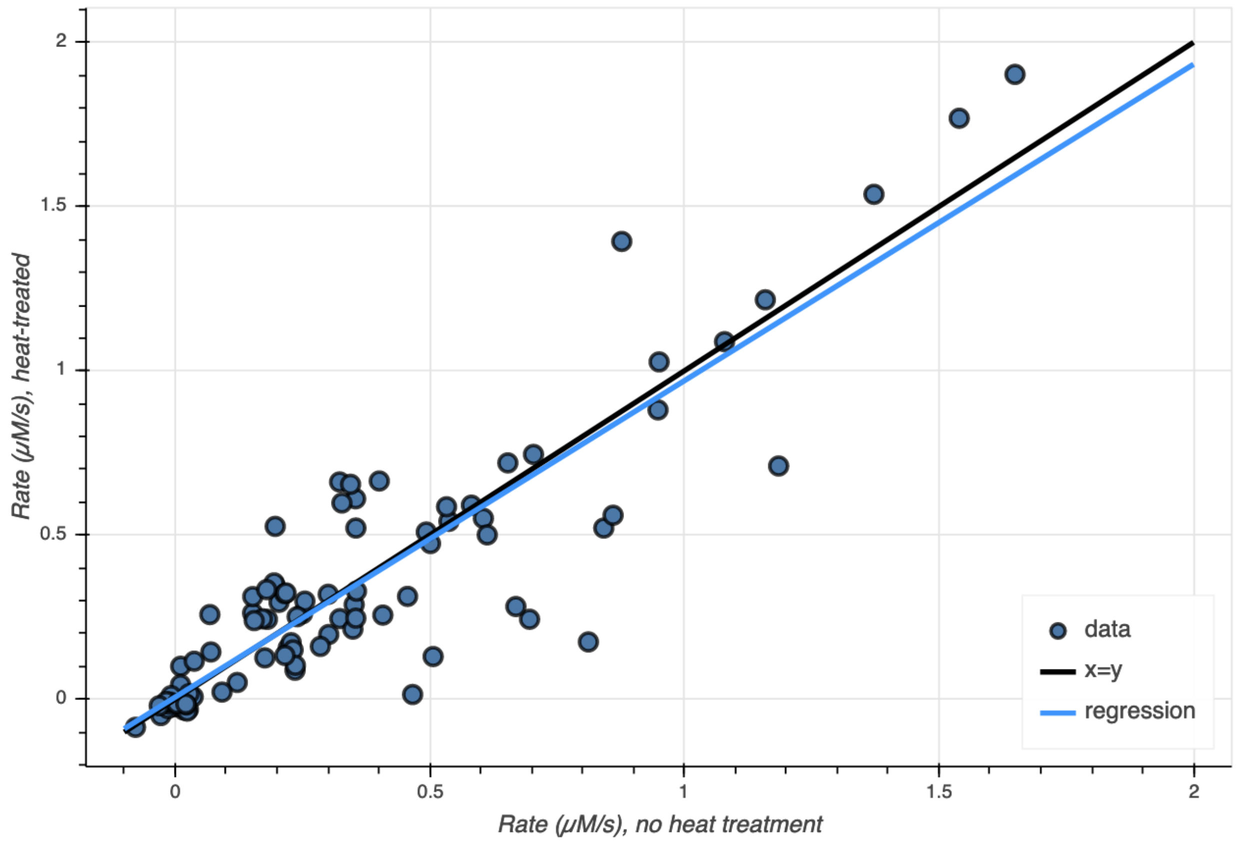

Supplementary Fig. 15 | Correlation between Trp formation by lysates with and without heat treatment. Trp formation by each OrthoRep-evolved variant from variant set 2, WT-003-1-A, Q90*-003-1-A, and Tri-100-2-A with or without heat treatment at 625 µM indole and 25 mM serine, evaluated by UV-vis spectrophotometry. Each point represents the initial rate of Trp formation by cell lysate generated via both heat treatment (1 hour at 75 °C) (y-axis) and a more mild method (x-axis) (see **Methods**). Linear regression on these data demonstrates a slope of 0.96, suggesting a negligible systematic decrease in activity with heat treatment across variants.
