# Supplementary material for: Scalable continuous evolution for the generation of diverse enzyme variants encompassing promiscuous activities
Source: Nat Commun. 2020 Nov 6;11:5644. doi: 10.1038/s41467-020-19539-6 (PMC7648111; doi:10.1038/s41467-020-19539-6)
Supplement: Supplementary file 6 — Description of Additional Supplementary Files [file 41467_2020_19539_MOESM6_ESM.pdf]

Title: Supplementary Data 1
Description: Plasmids used in Rix et al. 2020.

Title: Supplementary Data 2
Description: Primers used in Rix et al. 2020.

Title: Supplementary Data 3
Description: Yeast strains used in Rix et al. 2020.

Title: Supplementary Data 4
Description: Mutations and identification information for all individual TmTrpB sequences used in Rix et al. 2020.
